# MALDI-TOF mass spectrometry combined with machine learning algorithms to identify protein profiles related to malaria infection in human sera from Côte d’Ivoire

**DOI:** 10.1186/s12936-025-05362-1

**Published:** 2025-04-18

**Authors:** Fateneba Kone, Lucie Conrad, Jean T. Coulibaly, Kigbafori D. Silué, Sören L. Becker, Brama Kone, Issa Sy

**Affiliations:** 1 Institute of Medical Microbiology and Hygiene, Saarland University, Homburg, Germany; 2 UFR Biosciences, Université Félix Houphouët-Boigny, Abidjan, Côte d’Ivoire; 3 Centre Suisse de Recherches Scientifiques en Côte d’Ivoire, Abidjan, Côte d’Ivoire; 4 Swiss Tropical and Public Health Institute, Allschwil, Switzerland; 5 University of Basel, Basel, Switzerland; 6 Helmholtz Institute for Pharmaceutical Research Saarland, Saarbrücken, Germany; 7 Université Péléforo Gon Coulibaly, Korhogo, Côte d’Ivoire

**Keywords:** Malaria identification, *Plasmodium falciparum*, Serum, Matrix-assisted laser desorption/ionization-time of flight (MALDI-TOF) mass spectrometry, Machine learning (ML)

## Abstract

**Background:**

In sub-Saharan Africa, *Plasmodium falciparum* is the most prevalent species of malaria parasites. In endemic areas, malaria is mainly diagnosed using microscopy or rapid diagnostic tests (RDTs), which have limited sensitivity, and microscopic expertise is waning in non-endemic regions. Matrix-assisted laser desorption/ionization time-of-flight (MALDI-TOF) mass spectrometry (MS) is nowadays the standard method in routine microbiology laboratories for bacteria and fungi identification in high-income countries, but is rarely used for parasite detection. This study aims to employ MALDI-TOF MS for identifying malaria by distinguishing *P. falciparum*-positive from *P. falciparum*-negative sera.

**Methods:**

Sera were obtained from 282 blood samples collected from non-febrile, asymptomatic people aged 5 to 58 years in southern Côte d’Ivoire. Infectious status and parasitaemia were determined by both RDTs and microscopy, followed by a categorization into two groups (*P. falciparum*-positive and *P. falciparum*-negative samples). MALDI-TOF MS analysis was carried out by generating protein spectra profiles from 131 *Plasmodium*-positive and 94 *Plasmodium*-negative sera as the training set. Machine learning (ML) algorithms were employed for distinguishing *P. falciparum*-positive from *P. falciparum*-negative samples. Subsequently, a subset of 57 sera (42 *P. falciparum-*positive and 15 *P. falciparum-*negative) was used as the validation set to evaluate the best two of the four models trained.

**Results:**

MALDI-TOF MS was able to generate good-quality spectra from both *P. falciparum*-positive and *P. falciparum*-negative serum samples. High similarities between the protein spectra profiles did not allow for distinguishing the two groups using principal component analysis (PCA). When four supervised ML algorithms were tested by tenfold cross-validation, *P. falciparum*-positive sera were discriminated against *P. falciparum*-negative sera with a global accuracy ranging from 73.28% to 81.30%, while sensitivity ranged from 70.23% to 83.97%. The independent test performed with a subset of 57 serum samples showed accuracies of 85.96% and 89.47%, and sensitivities of 90.48% and 92.86%, respectively, for LightGBM and RF.

**Conclusion:**

MALDI-TOF MS combined with ML might be applied for detection of protein profiles related to *P. falciparum* malaria infection in human serum samples. Additional research is warranted for further optimization such as specific biomarkers detection or using other ML models.

**Graphical Abstract:**

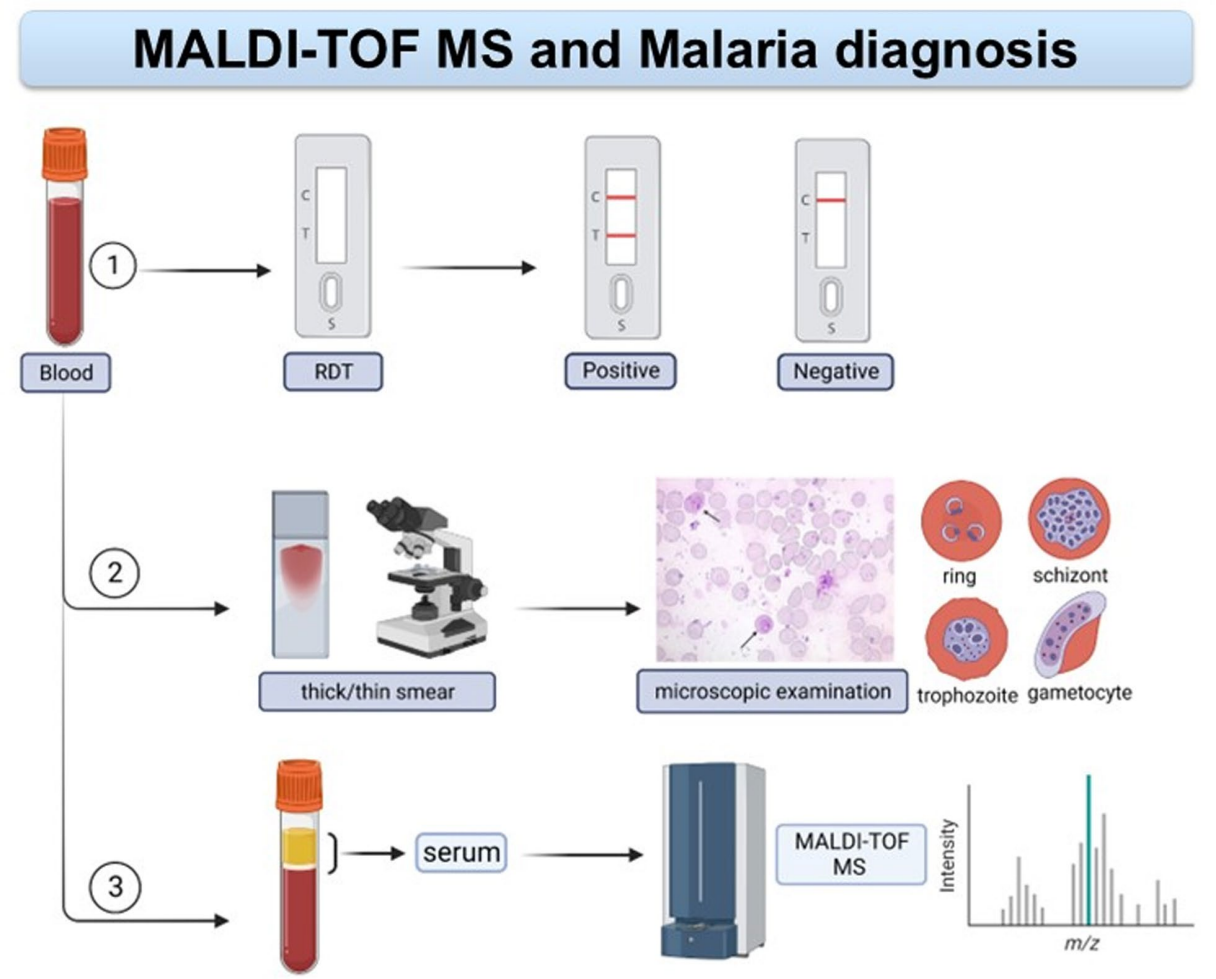

**Supplementary Information:**

The online version contains supplementary material available at 10.1186/s12936-025-05362-1.

## Background

Malaria is a protozoan disease caused by parasites of the genus *Plasmodium*, which are transmitted by *Anopheles* mosquitoes [1]. Species differentiation is of great importance for clinical decision-making and selection of the adequate treatment regimen [2]. It is the most important parasitic disease causing around 249 million cases in 85 malaria-endemic countries and 608,000 related deaths in 2022 [3]. The African region, especially sub-Saharan Africa, is the most affected region with 233 million cases, representing 94% of global cases and an estimated 580,000 annual deaths in 2022, most of which are attributable to *Plasmodium falciparum* [3, 4]. In Cote d’Ivoire, it is estimated that about 7,343,000 million malaria cases and 1555 deaths occur each year [3]. Infections and malaria-related deaths affect mostly pre-school-aged children (pre-SAC) (i.e., children under 5 years old), who are the most vulnerable populations (76% of global deaths) [3].

Malaria is mainly diagnosed by morphologic analysis of stained blood smears (thin and thick smears) and species differentiation is based on distinct morphological features, such as Schüffner’s dots, Maurer’s clefts, ring forms, shape of gametocytes [5]. Rapid diagnostic tests (RDTs) are also commonly employed, but their sensitivity varies and is lower for species other than *P. falciparum* [6]. Alternative methods such as nucleic acid amplification tests (NAATs) [e.g., real-time PCR, nested PCR, multiplex PCR, loop-mediated isothermal amplification (LAMP), or nucleic acid sequence-based amplification (NASBA)] based on the detection of 18S rRNA or other specific gene targets [e.g., telomere-associated repetitive element 2 (TARE-2), var acidic terminal sequence (varATS), mitochondrial DNA, or cytochrome oxidase III (COX-III)] can also be employed [7, 8]. The limitations of currently available techniques (e.g., the limited sensitivity of RTDs, challenging and sometimes unclear microscopy, and the scarcity of specific PCR kits in resource-limited countries, as well as the waning parasitological expertise in routine microbiology laboratories in non-endemic regions) are encouraging the development of new alternative approaches [8].

Matrix-assisted laser desorption/ionization time-of-flight (MALDI-TOF) mass spectrometry (MS) is a rapid, accurate, and cost-effective technique based on the analysis of protein spectra profiles. Indeed, it is nowadays used as the standard diagnostic method for the identification of bacteria, mycobacteria, and fungi in clinical laboratories in high-income countries [9–12]. The applicability of this technique has been investigated as a new tool for the identification of parasites, particularly for malaria diagnosis through haemozoin detection [13, 14]. More recently, a proof-of-concept study has investigated the utilization of MALDI-TOF MS as a new technique for diagnosing malaria by detecting *P. falciparum* isolated from infected red blood cells (iRBCs) using a culture-based approach [15]. The new trend of introducing artificial intelligence (AI), machine learning (ML), and deep learning (DL) into microbiology laboratories has demonstrated its applicability for improved diagnostic accuracy and automating the microscopic analysis of blood smears to detect malaria [16–18]. Similarly, the use of such approaches combining ML algorithms and MALDI-TOF MS could extend the applicability of MALDI-TOF MS to the direct analysis of more complex matrices such as blood, or serum for malaria diagnosis. Hence, a detailed assessment of MALDI protein spectra profiles of infected serum samples could be of a great interest in detecting *P. falciparum* malaria. Against this background, this study aimed to employ MALDI-TOF MS coupled with ML algorithms for the identification of *P. falciparum* malaria infection in human sera by assessing its identification accuracy for differentiating human serum of non-*P. falciparum* infected participants from serum infected with *P. falciparum*.

## Methods

### Ethics statement

All the procedures involving humans were conducted in strict accordance with the Institutional local Guidelines and approved by the National Ethical Committee for Life Science and Health of the Ministry of Health and Public Hygiene of Côte d’Ivoire under the number 056-21/MSHP/CNESVS-km. Consents were obtained from all adult participants as well as from parents or legal guardians of participants aged below 18 years. Clinical malaria cases were provided with medication in accordance with the country guidelines.

### Origin of samples

**Fig. 1:**
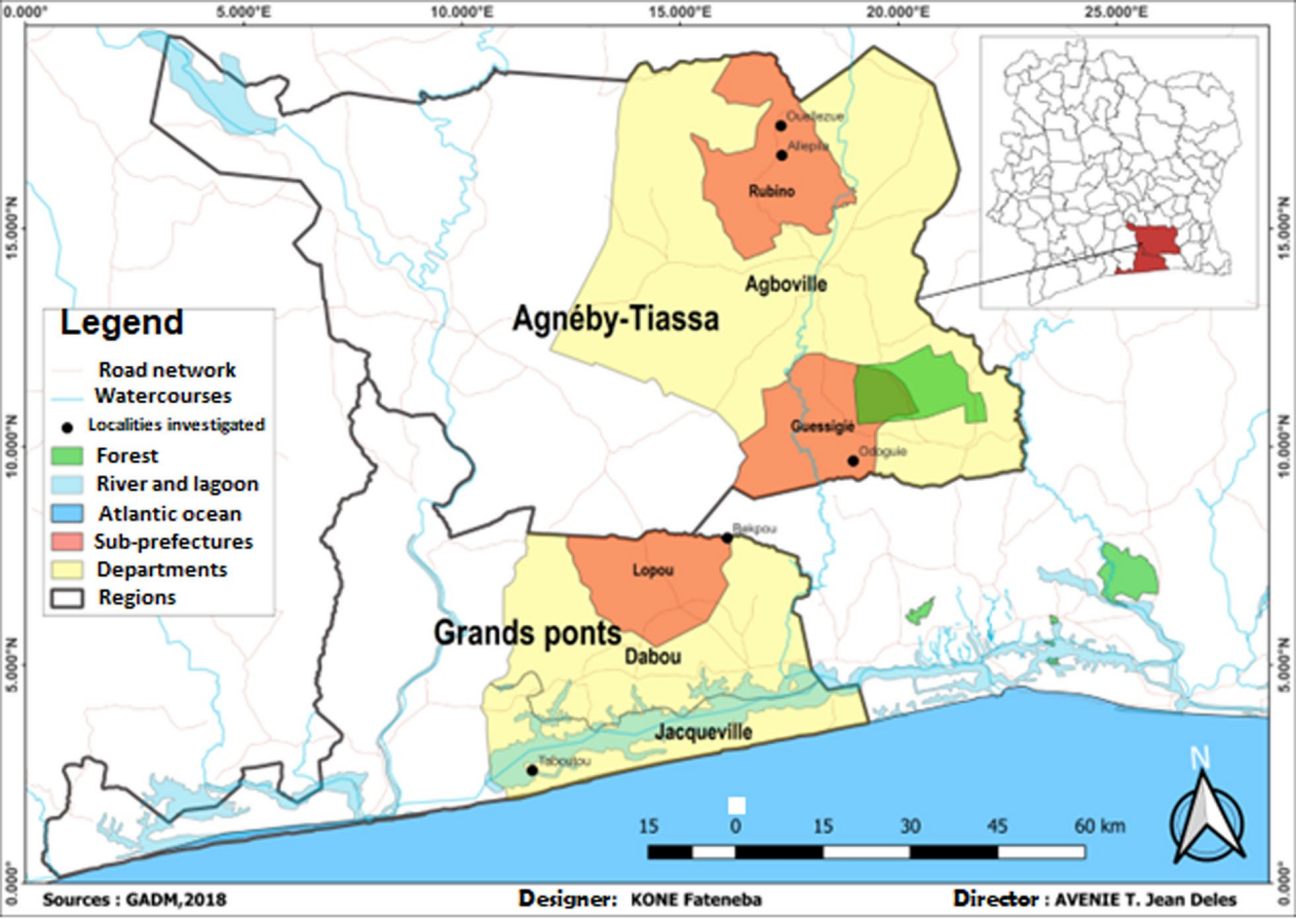
Map showing the study area: localization of the five malaria-endemic villages (Ouellezue, Allepila, Odoguie, Bekpou, and Taboutou) in Southern Côte d’Ivoire

Only asymptomatic participants were included. Individuals with an acute or uncontrolled illness (for example severe anaemia defined as haemoglobin < 8.0 d/dL, or persistent fever) assessed by a clinician, or who had received anthelminthics in the last four weeks, were excluded. Venous (for serum) and finger prick blood (for RDT) samples were obtained from consenting participants and/or with the consent of the parents/tutors aged between five and 58 years old in five malaria-endemic areas (Bekpou, Taboutou, Allepila, Ouellezue, and Odoguie) in the southern region of Côte d’Ivoire between November and December 2021 (Fig. 1). Fingerprick blood was used to perform the RDT and blood smear on-site and the venous blood samples collected were transported to the local laboratory for serum isolation.

### Antigenic and microscopic identification

RDTs (CareStart pLDH(pan), AccessBio, Somerset, NJ, USA, catalog number RMNM-02571) and blood smear techniques (thin and thick smears) were performed using finger prick blood samples following the manufacturer’s instructions to determine the infectious status, the parasite density, and the causative malaria infection. Parasite density was calculated using only thick blood smear microscopy readings. Individual parasitaemia was determined by assuming a standard white blood cells (WBCs) count of 8000/µL of blood [19]. Both asexual and gametocyte stages of the parasite were counted, in parallel with WBCs, until reaching 200 WBCs, or 500 WBCs if less than 10 parasites were detected [20]. A slide was considered negative if no asexual or gametocyte stage was found after counting 500 WBCs. Serum samples were subsequently prepared with the venous blood by centrifugation (5634×*g* for 10 min) and frozen in cryotubes before being transferred to the Institute of Medical Microbiology and Hygiene in Germany for further MALDI-TOF MS analysis. Upon receipt in Homburg (Germany), all samples were kept at –20 °C pending further examination.

### MALDI-TOF MS analysis

#### Sample preparation and protein extraction

A total of 282 serum samples selected accordingly to the positive RDTs and microscopy results (i.e., only *P. falciparum,* positive for both techniques), were defrosted at room temperature under a biosafety cabinet and measured by MALDI-TOF MS between April and Mai 2022. A protein extraction protocol using formic acid and acetonitrile described elsewhere was adapted and employed in this study [21, 22]. In brief, 50 µL of serum was placed in a tube containing a pinch of Zirconium beads and mixed by vortex. After centrifugation at 18,312 × g for 2 min, the supernatant was discarded and 300 µL of HPLC-grade water and 900 µL Absolut ethanol were added to the pellet (~ 10 µL of serum + beads) and mixed, followed by another centrifugation step at 18,312×*g* for 2 min. After discarding the supernatant, 10 µL of 70% (v/v) formic acid and 10 µL acetonitrile were added and mixed by vortex. This procedure was applied in triplicates (i.e., three different days) to each serum sample to ensure reproducibility.

#### MALDI-target plate preparation and measurements

The protein extracts obtained from the previous section were centrifuged at 18,312×*g* for 2 min. For each replicate, one µL of the clear supernatant was spotted four different spots onto the MALDI-TOF MS target plate (Bruker Daltonics, Bremen, Germany) then allowed to dry completely before overlaying 1 µL of α-cyano-4-hydroxycinnamic acid (CHCA) matrix solution (Bruker Daltonics, Bremen, Germany), composed of saturated CHCA 50% (v/v) of acetonitrile, 2.5% (v/v) of trifluoroacetic acid and 47.5% (v/v) of LC–MS grade water. Each of the four spots was then measured two times to obtain 8 spectra per protein extraction (i.e., 24 spectra per sample). Of note, not all samples in one category were acquired separately from the other category. Simultaneous acquisition has also occurred on the same plate. A total of 173 *P. falciparum*-positive, and 109 *P. falciparum-*negative sera were analysed using the FlexControl® software version 3.4 (Bruker Daltonics, Bremen, Germany) for spectra acquisition. Bacterial test standard (BTS) (Bruker Daltonics, Bremen, Germany), which is an extract of *Escherichia coli* that is spiked with two high molecular weight proteins, was used to calibrate the mass spectrometer. Dried MALDI plates were then placed into the Microflex LT Mass Spectrometer (Bruker Daltonics) for measurements.

#### MALDI-TOF MS parameters

Measurements were performed using the AutoXecute algorithm implemented in the FlexControl^©^ software version 3.4. For each spot, a total of 240 laser shots (40 shots each, in six random positions) were carried out automatically to generate protein mass profiles in linear positive ion mode with a laser frequency of 60 Hz, a high voltage of 20 kV, a pulsed ion extraction of 180 ns, a total of at least 80 shots allowed before automated termination, an evaluation mass range of ± 200 Da, and a minimum intensity threshold of 600 a.u. Mass charge ratios range (m/z) were measured between 2 and 20 kDa.

#### Spectra treatment, classification and comparison analysis

##### Pre-processing parameters

A total of 6768 (282 samples × 3 biological replicates × 8 technical replicates) raw spectra were generated. The dataset was randomly split into training (i.e., 80% of the data, including 131 *P. falciparum*-positive, and 94 *P. falciparum*-negative sera) and test (i.e., 20% of the data, including 42 *P. falciparum*-positive, and 15 *P. falciparum*-negative sera) sets using Python (version 3.13.1) and exported into the online Clover MS Data Analysis® software (https://platform.clovermsdataanalysis.com, Clover Bioanalytical Software, Granada, Spain) for further investigation. Default parameters reported elsewhere were used during preprocessing [23, 24]. Variance stabilization and Savitzky–Golay filter (window length 11; polynomial order 3) were applied for smoothing spectra, the baseline was removed using the top-hat filter method (factor 0.02). Replicated spectra were aligned using the following parameters: allowed shift, medium; constant tolerance, 0.2 Da; linear tolerance, 2000 ppm in order to create one average spectrum per sample, which will be used for classification and comparison analysis.

##### Classification: training using tenfold cross-validation of four ML algorithms

A peak matrix was generated from pre-processed spectra and used for comparison analysis. Spectra were normalized using Total Ion Current (TIC) normalization, followed by a “threshold method” (factor 0.01), where peaks with an intensity below 1% of the maximum intensity were not included; a constant tolerance of 0.5 Da; and a linear tolerance of 500 ppm [23, 24].

The spectra were classified into two categories (positive and negative) according to the infectious status and subjected to different classification algorithms in order to distinguish *P. falciparum-*positive sera from *P. falciparum*-negative sera*.* Supervised (i.e., a guided learning system with training data associated with the pre-defined labels) ML algorithms were used to assess the classification. Four widely used supervised ML algorithms for MALDI-TOF mass spectra analysis [linear super vector machine (SVM) with PCA technique applied using 87 components (95.16% variance), partial least squares-discriminant analysis (PLS-DA) with PCA technique applied using 9 components (43.95% variance), random forest (RF), and light gradient boosting model (GBM)] were applied [25, 26]. These algorithms were trained using k-fold cross-validation (k = 10) for internal validation. Oversampling was also applied using SMOTE algorithm. A confusion matrix (generating values such as accuracy, specificity, sensitivity, F1-score, positive prediction value or precision, and negative prediction value), as well as the area under receiver operating characteristic (AUROC) curve, were used as performance metrics of the supervised ML algorithms to evaluate their discrimination power, with higher values indicating greater discrimination [26].

##### Independent test of the two best models

The two best models of the four tested during the training phase were evaluated by testing MALDI-spectra generated from a subset of 57 serum samples (42 positives and 15 negatives). The spectra were pre-processed using the same parameters as the training phase.

## Results

### Prevalence and malaria confirmation

**Fig. 2:**
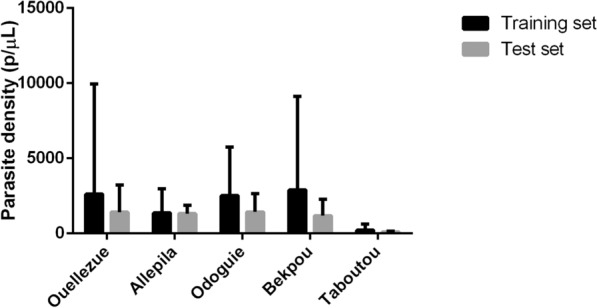
Average parasite density (p/µL) determined by blood smear in the five malaria-endemic villages (Ouellezue, Allepila, Odoguie, Bekpou, and Taboutou) in Southern Côte d’Ivoire

A total of 486 individuals aged between five and 58 years old (mean = 11.2 ± 5.1) participated in this study, including 270 males, 214 females, and 2 people for whom gender information was not reported. RDT and microscopy analyses showed *P. falciparum* infection prevalences of 69.3% (337/486) and 65.6% (319/486) respectively. Most of the positive samples were *P. falciparum* (95.6%; 305/319) with parasite densities ranging between 48 and 34′320 p/µL. *Plasmodium malariae* and mixed *P. malariae/falciparum* were detected in 6/319 (1.9%) and 8/319 (2.5%) positive samples, respectively (Fig. 2; Additional file).

### Spectra analysis and cross-validation performance

**Fig. 3:**
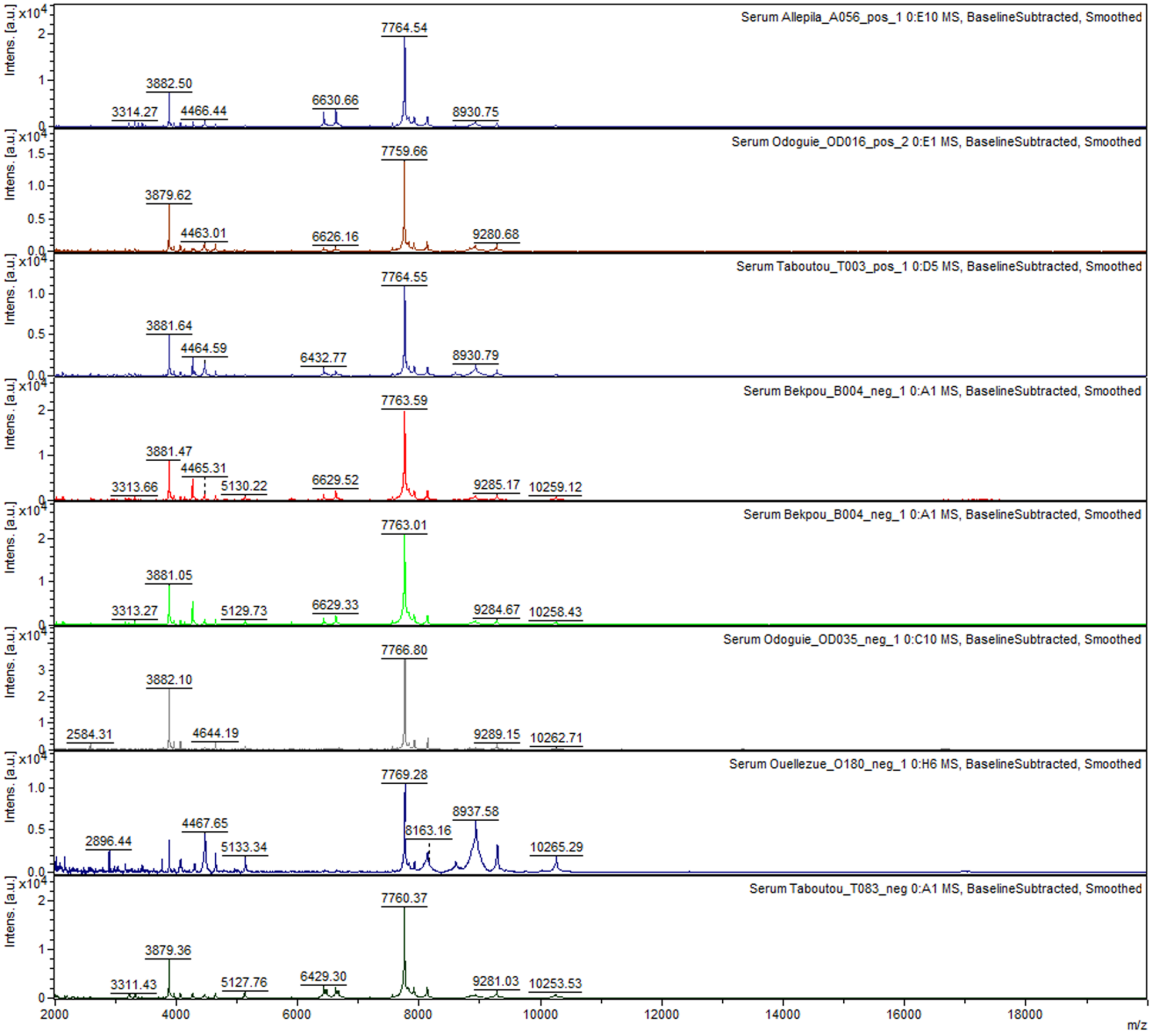
Representative protein spectra profiles of *P. falciparum*-positive and *P. falciparum*-negative sera

Good quality spectra with high intensities were generated for 173 *P. falciparum-positive* (131 training, and 42 test) and 109 *P. falciparum*-negative (94 training, and 15 test) sera. Spectra profiles of both groups are very similar with many common peaks (Fig. 3)*.*

**Fig. 4:**
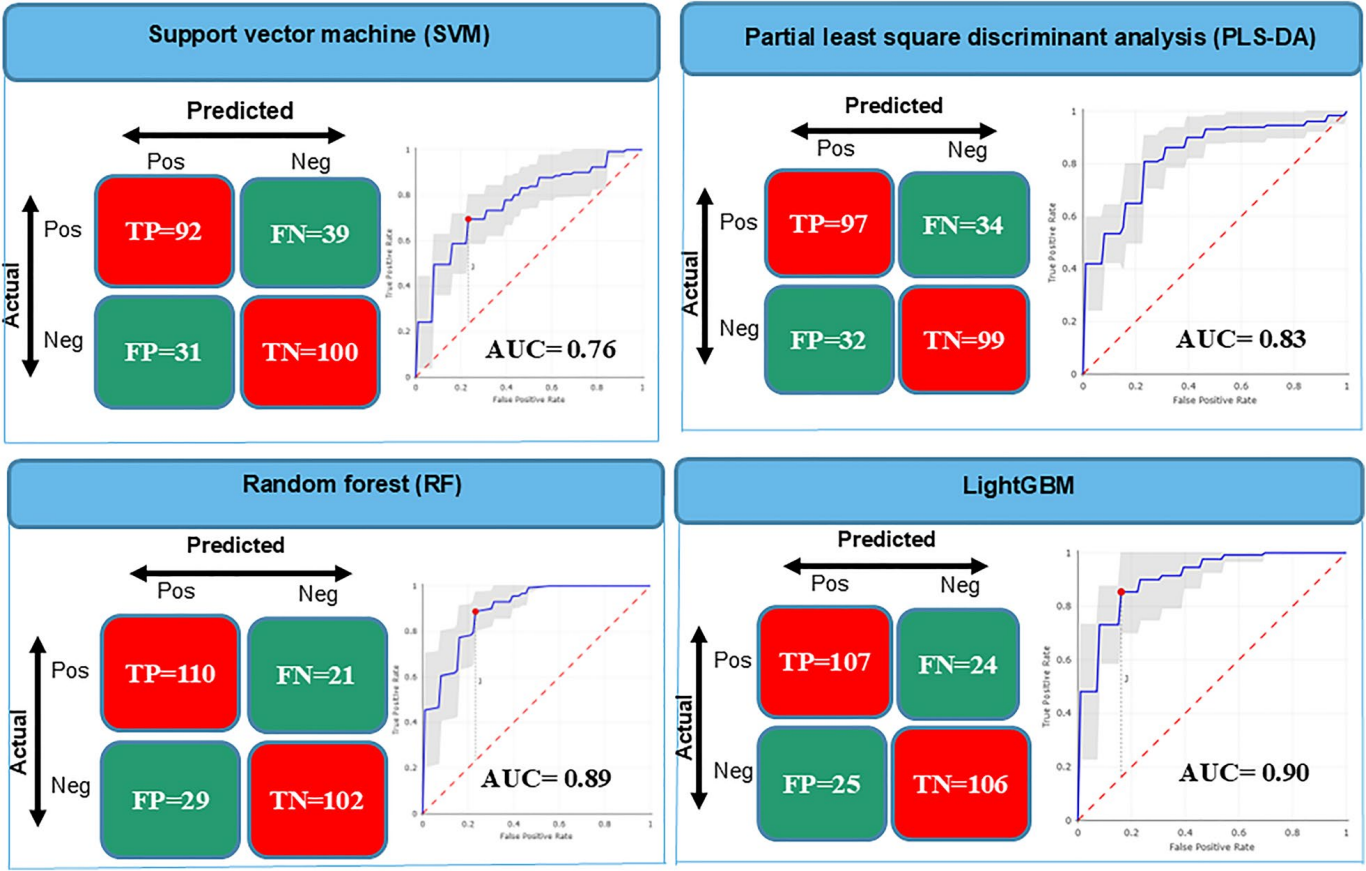
Confusion matrix and ROC curves showing the classification results of *P. falciparum*-positive and *P. falciparum*-negative sera using MALDI-TOF MS spectra and supervised ML algorithms. Pos: positive; Neg: negative; TP: true positive; TN: true negative; FP: false positive; FN: false negative; ROC: receiver operating characteristic; AUC: area under the curve

**Fig. 5:**
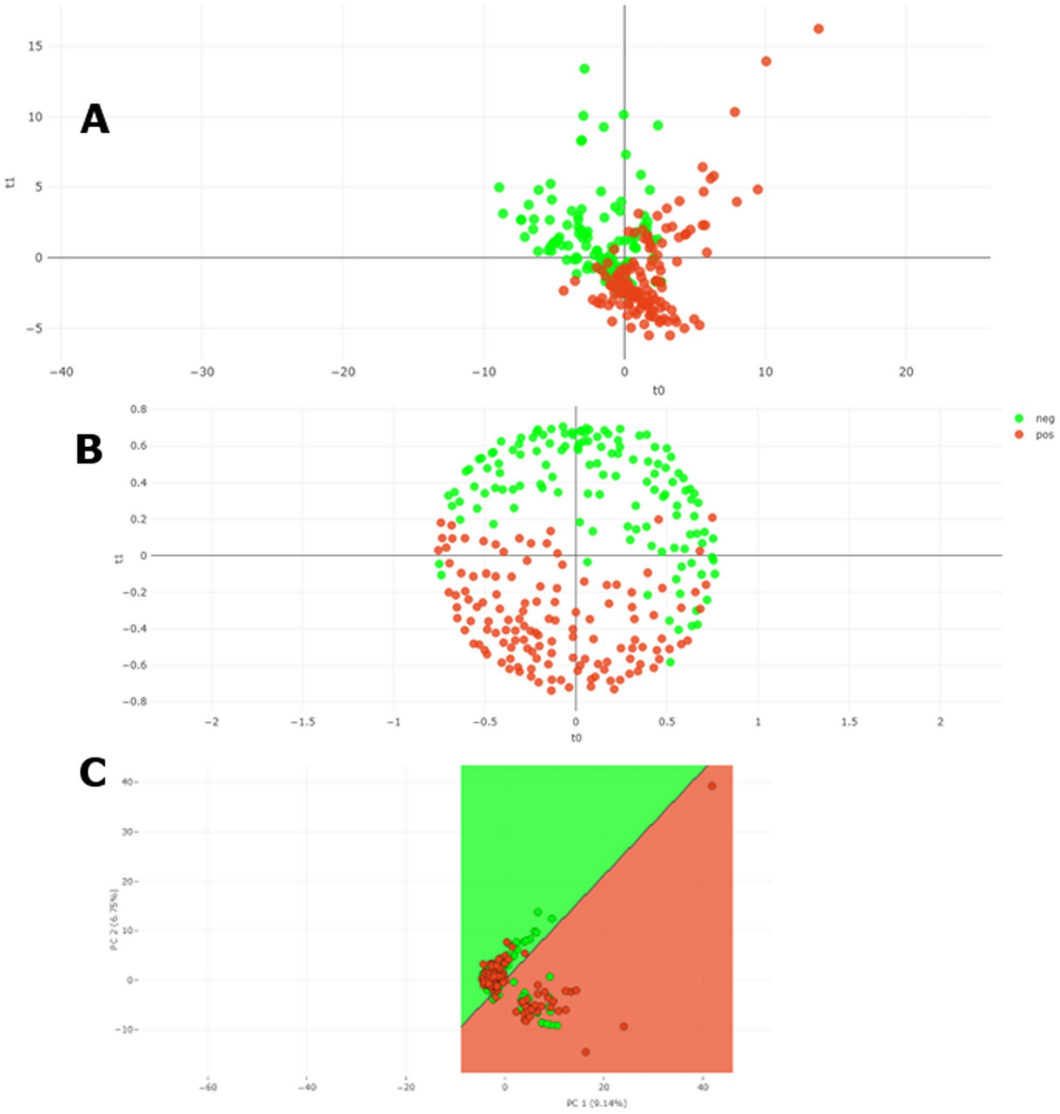
Distance plots of tenfold cross-validation results using supervised ML algorithms in Clover MS Data Analysis® software, based on peak matrix generated with a threshold of 1% and TIC normalization: distance plots of positive and negative spectra. **A** Two-dimensional view of partial least squares-discriminant analysis (PLS-DA); **B** Two-dimensional view of Random Forest (RF); **C** Two-dimensional view of linear support vector machine (SVM). Pos: positive (in red); Neg: negative (in green)

When supervised ML algorithms were employed, tenfold cross-validation results for positive sera showed accuracies of 81.68% (107/131) for LightGBM, 83.97% (110/12) for RF, 74.05% (97/131) for PLS-DA, and 70.23% (92/131) for SVM. For negative sera, accuracies were 80.92% (106/131), 77.86% (102/131), 75.57% (99/131), and 76.34% (100/131), respectively for LightGBM, RF, PLS-DA, and SVM. Global accuracies were 81.3%, 80.92%, 74.81%, and 73.28%, respectively for LightGBM, RF, PLS-DA, and SVM. Other metrics such as sensitivity, specificity, and F1-score (harmonic mean of precision and sensitivity) showed higher values for LightGBM (sensitivity: 81.68%; specificity: 80.92%, F1-score: 81.37%) and RF (sensitivity: 83.97; specificity: 77.86%, F1-score: 81.48%) than PLS-DA (sensitivity: 74.05%; specificity: 75.57%, F1-score: 74.62%) and SVM (sensitivity: 70.23%; specificity: 76.34%, F1-score: 72.44) (Table 1). The receiver operating curves (ROC), indicating the statistical significance had area under the curve (AUC) values of 0.90, 0.89, 0.83, and 0.76 for LightGBM, RF, PLS-DA, and SVM, respectively (Fig. 4). Distance plots visualization of the different algorithms confirmed the performance metrics as the separation of the clusters is clearer with RF than with PLS-DA and SVM (Fig. 5).

**Table 1:** Tenfold cross-validation results showing discrimination between *P. falciparum-positive sera* from *P. falciparum-negative* sera with *P. falciparum-positive* sera considered as positive category: scores (in %) obtained with four different classifying algorithms (SVM, PLS-DA, RF, and LightGBM)

	Accuracy %	Sensitivity %	Specificity %	Error rate %	PPV %	NPV %	F1-score %
SVM	73.28	70.23	76.34	26.72	74.8	71.94	72.44
PLS-DA	74.81	74.05	75.57	25.19	75.19	74.44	74.62
RF	80.92	83.97	77.86	19.08	79.14	82.93	81.48
LightGBM	81.3	81.68	80.92	18.7	81.06	81.54	81.37

PPV: positive predictive values; NPV: negative predictive values

### Independent test of the ML-based models

**Fig. 6:**
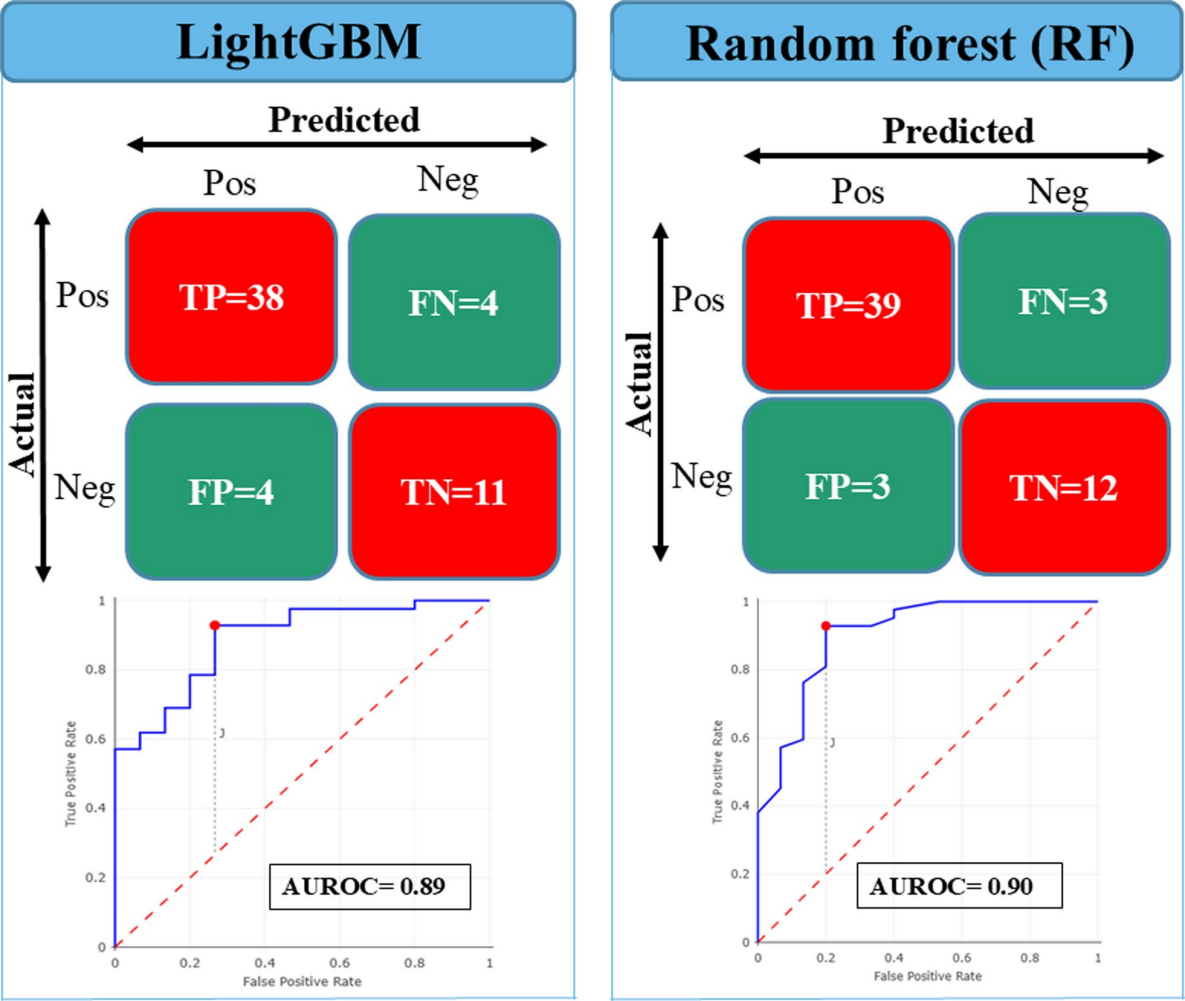
Confusion matrix and ROC curves showing the independent test results of *P. falciparum*-positive and *P. falciparum*-negative sera using MALDI-TOF MS spectra and supervised ML algorithms (LightGBM, and RF). Pos: positive; Neg: negative; TP: true positive; TN: true negative: FP: false positive; FN: false negative; AUC: area under the curve

The best two ML-based models (Light GBM and RF) of the training phase tested with a subset of 57 sera samples (42 positives and 15 negatives) showed accuracies of 90.48% (38/42), and 73.33% (11/15) respectively for positive and negative, for LightGBM algorithm. For the RF algorithm, accuracy rates were 92.86% (39/42), and 80% (12/15) for positive and negative sera, respectively (Fig. 6). Globally, these two models have accuracy rates of 85.96%, and 89.47% respectively for LightGBM and RF. While specificity performances were 73.33% for LightGBM, and 80% for RF (Table 2).

**Table 2:** Independent test results of two ML algorithms (LightGBM, and RF) showing discrimination performances between *P. falciparum-positive* sera from *P. falciparum-negative* sera with *P. falciparum-positive* sera considered as positive category

	Accuracy %	Sensitivity %	Specificity %	Error rate %	PPV %	NPV %	F1-score %
LightGBM	85.96	90.48	73.33	14.04	90.48	73.33	90.48
RF	89.47	92.86	80	10.53	92.86	80	92.86

LightGBM: light gradient boosting model; RF: random forest; PPV: positive predictive values; NPV: negative predictive values

## Discussion

MALDI-TOF MS is nowadays a widely used technique in clinical routine microbiology laboratories in high-income countries [27]. Recently, new applications (e.g., parasite identification) have also been investigated, but have yet to be applied in clinical samples [15, 28]. Combined with ML, MALDI-TOF MS enables a more in-depth study (e.g., comparative analysis of infected vs uninfected specimens), especially for unpurified or complex biological matrices such as blood or serum samples, represents a step forward in the quest for new, clinically applicable diagnostic methods.

In this study, MALDI-TOF MS associated with ML algorithms allowed distinguishing *P. falciparum*-positive from *P. falciparum*-negative samples using protein spectra profiles generated from serum samples. Despite high similarities of the spectra profiles of both groups, trained classification models using supervised ML algorithms such as LightGBM and RF showed significant abilities to distinguish *P. falciparum*-positive from *P. falciparum*-negative sera with a global accuracy of 81.3%, and 80.9% for LightGBM and RF, respectively. In addition, the ability to correctly classify new independent sera reaches accuracies of 85.96%, and 89.47% for LightGBM and RF, respectively.

In the context of exploring alternative MALDI-based methods to improve malaria diagnosis, many other studies, particularly in entomology, have attempted to identify the different species of the parasite vector (i.e., *Anopheles* mosquitoes), the origin of their blood meal, and if they carry the malaria parasite or not [29–36].

Other studies have attempted malaria screening through the detection of haemozoin using laser desorption time-of-flight (LD-TOF) MS [13, 14, 37]. More recently, Stauning and colleagues have reported the detection of malaria using MALDI-TOF MS by targeting directly the *Plasmodium* parasite extracted from human blood [15]. The authors also reported about 30 *P. falciparum*-specific peaks, and none of them could be found in this present study due to the differences in the methodological approach. Indeed, Stauning and colleagues analyzed *P. falciparum* directly isolated from RBCs of human blood, while this study focused on *P. falciparum*-positive sera. As regards the data available to date, this is the first study to use MALDI-TOF MS combined with ML algorithms for identifying *P. falciparum*-infected human sera from endemic regions.

The present study is limited by the sample pre-processing and the type of material used (human serum), which will be more likely to display host and immune-related proteins, making it difficult to detect *Plasmodium*-specific peaks. Also, the negative group consisted of participants whose serum was negative for *P. falciparum*, but who were not tested for other infections (e.g., bloodstream infection), which could affect specificity if such infections occurred. Other limitations are the restricted origin of the samples (all sera were isolated from the same endemic country, Côte d’Ivoire), the limited size of the dataset, and the lack of species diversity leading to limited specificity results since the prediction models were trained with a positive category (i.e., positive sera) comprising only one type of species (i.e., *P. falciparum*). Nevertheless, *P. falciparum* is globally the most prevalent species in sub-Saharan Africa, but is also reported elsewhere outside Africa, having these ML-based models developed with such a dataset could be helpful in quickly identifying most malaria cases [3, 38]. However, to correctly deploy appropriate public health strategies based on reliable epidemiological data, and limit the severity and the spread of the parasite by ensuring effective treatment, it is crucial to precisely identify the parasite species [39, 40]. Hence, further investigations are needed (i.e., training more robust models with a dataset including all types of species from various regions in endemic countries) to optimize the method for better performance and enable *Plasmodium* species differentiation. Furthermore, employing alternative approaches such as deep learning (e.g., Siamese Neural Network (SNN), or Triplet Neural Networks (TNN)) combined with high-resolution proteomics would enable intensity variation analysis to detect potential specific biomarkers for malaria [41].

From a clinical diagnostic perspective, more effort should be dedicated to the direct application of MALDI-TOF MS on biological samples from humans (e.g., human blood and/or serum). Indeed, this could be achieved as has already been demonstrated for other diseases such as COVID-19, where plasma proteome fingerprint predicted high (hospitalized) and low-risk (outpatients) cases with 92% accuracy; or serum analysis of COVID-patients in comparison with control cases achieved 99% accuracy [42, 43]. Likewise, MALDI/ML analysis of serum protein fingerprints showed significant differences between liver cancer patients and healthy controls [44].

MALDI-TOF mass spectrometry offers many advantages, including the ability to carry out rapid, and reliable analyses. However, even if sample analysis is inexpensive, the acquisition price remains high (~ one hundred thousand euros) compared to microscopy, which is the gold standard for malaria diagnosis.

## Conclusion

The results of this study provide evidence of the general applicability of the MALDI-TOF MS**/**ML combination for the identification of *P. falciparum-*positive human serum stemming from endemic areas in Côte d’Ivoire. Further investigations are needed to optimize this method for better performance in terms of accuracy, sensitivity, and specificity, but also to enable species differentiation and quantification.

## Supplementary Information

Supplementary Material 1. Epidemiological data and *Plasmodium* prevalence using RDTs and microscopy.

## Data Availability

All data supporting the findings of this study are available within the paper and its Supplementary Information.

## Abbreviations

MALDI-TOF MS: Matrix-Assisted laser desorption/ionization-time of flight mass spectrometry
CHCA: α-Cyano-4-hydroxycinnamic acid
BTS: Bacterial test standard
ML: Machine learning
PCA: Principal component analysis
SVM: Support vector machine
PLS-DA: Partial least squares-discriminant analysis
RF: Random forest
Light GBM: Light gradient boosting model
ROC: Receiver operating characteristic
AUROC: Area under receiver operating characteristic
